# Cholangioscopy in IgG4-related sclerosing cholangitis using texture and color enhancement imaging and red dichromatic imaging

**DOI:** 10.1055/a-2155-4853

**Published:** 2023-08-30

**Authors:** Tomohiro Ishii, Takashi Kaneko, Ayumi Murakami, Masato Enomoto, Kazuya Sugimori, Ichiro Kawana, Shin Maeda

**Affiliations:** 1 Department of Gastroenterology, Saiseikai Yokohamashi Nanbu Hospital, Yokohama, Japan; 2 Gastroenterological Center, Yokohama City University Medical Center, Yokohama, Japan; 3 Department of Pathology, Saiseikai Yokohamashi Nanbu Hospital, Yokohama, Japan; 4 Department of Gastroenterology, Yokohama City University Graduate School of Medicine, Yokohama, Japan

Texture and color enhancement imaging (TXI) and red dichromatic imaging (RDI) modes are new image-enhancement options available on the EVIS X1 system (Olympus Corporation, Tokyo, Japan). TXI emphasizes slight differences in color tone and structural changes on the mucosal surface under normal light 1 , whereas RDI may improve the visibility of deep blood vessels 2 . We previously reported the use of TXI- and RDI-mode cholangioscopy (CHF-B290, Olympus Corporation) in cholangiocarcinoma 3 . Here, we describe this technique in a patient with IgG4-related sclerosing cholangitis (IgG4-SC) and autoimmune pancreatitis (AIP).

An 87-year-old woman presented with elevated liver enzymes and diffuse pancreatic enlargement ( Fig. 1 , Fig. 2 ). Endoscopic retrograde cholangiopancreatography (ERCP) revealed segmental stenosis of the distal bile duct, preventing passage of the cholangioscope. A biliary stent was placed. Histopathology revealed no malignant findings; high IgG and IgG4 levels led to the diagnosis of AIP + IgG4-SC. Due to the patient’s age, stent replacement and stenosis reassessment were performed 2 months later without medication. The second ERCP showed slight improvement in stenosis, allowing passage of the cholangioscope. Cholangioscopy revealed partially dilated and slightly tortuous vessels without encasement or fusion 4 at the hilar bile duct. Dilated vessels were more clearly observed, and the fine surface structures of the thickened mucosa were more pronounced in TXI mode 1 than with white-light imaging. In RDI mode 3, the color of the bile disappeared and the dilated vessels were more strongly accentuated than with TXI. The mucosal surface was smooth, and no tumor vessels were observed at the stenosis ( Video 1 ). The addition of TXI and RDI to cholangioscopy may therefore aid the diagnosis of IgG4-SC. A third ERCP 4 months later showed slight bile duct stenosis that did not require stenting; no adverse events or recurrence of symptoms were observed over the 10-month follow-up period.

**Fig. 1:**
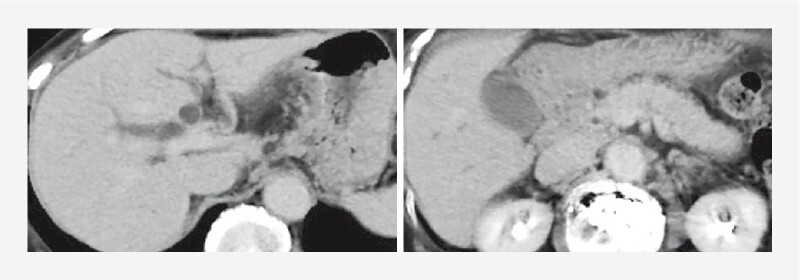
Abdominal contrast-enhanced computed tomography showed diffuse enlargement of the pancreas, distal bile duct stenosis, and intrahepatic bile duct dilation.

**Fig. 2:**
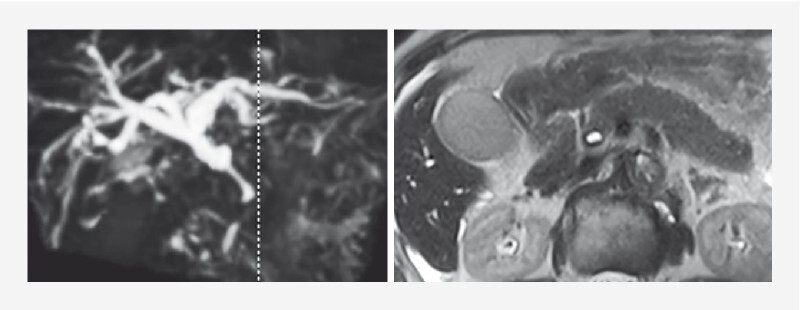
Magnetic resonance cholangiopancreatography showed intrahepatic bile duct dilatation, stenosis of the distal bile duct, and diffuse enlargement of the pancreas. The main pancreatic duct was poorly delineated.

**Video 1** Findings of the cholangioscopy using texture and color enhancement imaging mode 1 and red dichromatic imaging mode 3 in a patient with IgG4-related sclerosing cholangitis and autoimmune pancreatitis.


Endoscopy_UCTN_Code_TTT_1AR_2AG

## Notes

Endoscopy E-Videos

https://eref.thieme.de/e-videos
*E-Videos* is an open access online section of the journal *Endoscopy* , reporting on interesting cases and new techniques in gastroenterological endoscopy. All papers include a high-quality video and are published with a Creative Commons CC-BY license. Endoscopy E-Videos qualify for HINARI discounts and waivers and eligibility is automatically checked during the submission process. We grant 100% waivers to articles whose corresponding authors are based in Group A countries and 50% waivers to those who are based in Group B countries as classified by Research4Life (see: https://www.research4life.org/access/eligibility/ ). This section has its own submission website at https://mc.manuscriptcentral.com/e-videos
